# Assessing Fine-Granularity Structural and Functional Connectivity in Children With Attention Deficit Hyperactivity Disorder

**DOI:** 10.3389/fnhum.2020.594830

**Published:** 2020-11-13

**Authors:** Peng Wang, Xi Jiang, Hanbo Chen, Shu Zhang, Xiang Li, Qingjiu Cao, Li Sun, Lu Liu, Binrang Yang, Yufeng Wang

**Affiliations:** ^1^Peking University Sixth Hospital, Institute of Mental Health, Beijing, China; ^2^National Clinical Research Center for Mental Disorders and the Key Laboratory of Mental Health, Ministry of Health (Peking University), Beijing, China; ^3^Shenzhen Children’s Hospital, Shenzhen, China; ^4^School of Life Sciences and Technology, MOE Key Lab for Neuroinformation, University of Electronic Science and Technology of China, Chengdu, China; ^5^Cortical Architecture Imaging and Discovery Lab, Department of Computer Science and Bioimaging Research Center, University of Georgia, Athens, GA, United States; ^6^School of Computer Science, Northwestern Polytechnical University, Xi’an, China; ^7^Massachusetts General Hospital, Harvard Medical School, Boston, MA, United States

**Keywords:** attention deficit hyperactivity disorder, fMRI, resting state, functional connectivity, diffusion tensor imaging, structural connectivity

## Abstract

Attention deficit hyperactivity disorder (ADHD) was considered to be a disorder with high heterogeneity, as various abnormalities were found across widespread brain regions in recent neuroimaging studies. However, remarkable individual variability of cortical structure and function may have partially contributed to these discrepant findings. In this work, we applied the Dense Individualized and Common Connectivity-Based Cortical Landmarks (DICCCOL) method to identify fine-granularity corresponding functional cortical regions across different subjects based on the shape of a white matter fiber bundle and measured functional connectivities between these cortical regions. Fiber bundle pattern and functional connectivity were compared between ADHD patients and normal controls in two independent samples. Interestingly, four neighboring DICCCOLs located close to the left parietooccipital area consistently exhibited discrepant fiber bundles in both datasets. The left precentral gyrus (DICCCOL 175, BA 6) and the right anterior cingulate gyrus (DICCCOL 321, BA 32) had the highest connection number among 78 pairs of abnormal functional connectivities with good cross-sample consistency. Furthermore, abnormal functional connectivities were significantly correlated with ADHD symptoms. Our studies revealed novel fine-granularity structural and functional alterations in ADHD.

## Introduction

Attention deficit hyperactivity disorder (ADHD) is one of the most common neurobehavioral psychiatric disorders of childhood, and its prevalence rates based on teacher reports were an estimated 5.47% (Polanczyk et al., 2014). The typical symptoms are characterized as excessive inattention, hyperactivity/impulsiveness, or their combinations (Cortese, 2012). Over the past several decades, functional and structural neuroimaging data provided a promising opportunity to understand this disorder better by revealing various differences in brain regions between ADHD patients and controls (Albajara Saenz et al., 2019). The classical prefrontal–striatal model (Castellanos et al., 2006) of ADHD could be extended to include other neural circuits and their relationships from the perspective of large-scale brain networks (Bush, 2010; Castellanos and Proal, 2012). The studies of resting-state functional connectivity also highlight the importance of brain interval connection.

The scattered brain areas that exhibit neuroactivities at resting state are called default mode network (DMN), which includes medial prefrontal cortex, precuneus/posterior cingulate cortex, lateral parietal cortex, and inferior temporal lobule. Previous studies have shown that the synchronization of neural activity (functional connectivity; Friston et al., 1993) within the default network and between the default network and the task-positive network is closely related to cognitive function. For example, a stronger negative correlation between default network and frontal–parietal network is associated with better behavioral performance (Kelly et al., 2008). An experiment comparing 24-h sleep deprivation state with natural arousal state showed that sleep deprivation increased the variation of subjects’ reaction time in behavioral performance, and this phenomenon was related to the functional connectivity within DMN and the weakening of negative correlation between component DMN and external brain network (De Havas et al., 2012). Recently, studies using electroencephalogram have also proved the relationship between the variation of behavioral performance and the synchronization of brain activity (Gerrits et al., 2019; Machida et al., 2019; Kucyi et al., 2020). This association also exists in the ADHD population. In ADHD children, stronger frontal–striatal functional connectivity at the resting state is associated with better executive function, especially the inhibitory control (Li et al., 2014). Abnormal thalamo-caudate functional connectivity is associated with poor spatial working memory in ADHD children (Mills et al., 2012). In ADHD adults, functional connectivity within DMN and between DMN and attention network was associated with cognitive performance (Mowinckel et al., 2017).

A recent review also confirmed that the abnormal synchrony among brain regions in resting state is one of the characteristics of ADHD, mainly the decreasing synchronization between the anterior and posterior regions of DMN and the absence of anticorrelation between DMN and task-positive network (Posner et al., 2014; Castellanos and Aoki, 2016). In youths with ADHD, the anticorrelation between the dorsal anterior cingulate cortex (ACC) and DMN structures, including the posterior cingulate cortex, is decreased (Sun et al., 2012). Other studies with similar findings in the adult/child population (Sato et al., 2012; Hoekzema et al., 2014; Kumar et al., 2020). Furthermore, methylphenidate hydrochloride, as the most commonly used effective medication for the treatment of ADHD, can normalize the deactivation of the ventromedial prefrontal cortex (vmPFC) and posterior cingulate gyrus and improve cognitive performance (Peterson et al., 2009; Liddle et al., 2011). Multiple studies also confirmed that medication could normalize the DMN function connectivity of ADHD patients (Pereira-Sanchez and de Castro Manglano, 2017) and improve their cognitive task performance (Mowinckel et al., 2017). In conclusion, studying the relationship between brain regions from the whole-brain level, rather than a single region or network, is necessary for ADHD research.

However, in the ADHD research field, high heterogeneity has become an important issue (Luo et al., 2019). In addition to the complex etiology, individual differences in brain structure and function are also important factors. In the studies on the relationship between brain regions, how to define “brain regions” in different brains is a critical problem. The consensus is that the research results of functional connectivity based on seed points [regions of interest (ROIs)] are easily affected by the selection of seed points, which is a relatively subjective process (Power et al., 2012). Due to the possible heterogeneity inside the selected brain regions and the complexity of the human brain, the results may vary with the size of the selected “brain region.” Using an activation experiment to define ROI could reduce the feasibility and repeatability of the research. To define ROI by meta-analysis according to the ROIs in previous studies, we need to ignore the possible differences in brain structure among individuals, and previous experiments could also have the problem in selecting ROI. In the previous studies of functional connectivity, a very small change in the ROI will greatly change the results. This phenomenon is largely due to the limitations of functional magnetic resonance imaging (fMRI) technology (Zhu et al., 2011; Li et al., 2013).

Furthermore, regarding the future clinical application of resting-state fMRI (RS-fMRI), pointed out that one of the future directions would be big sample data, and acquiring large samples needs multisite data sharing. Big data from a single site may produce statistically significant but trivial results, whereas no result is believable until it has been replicated in multiple independent datasets (Castellanos et al., 2013). Big data would be a possible solution to the heterogeneity of ADHD fMRI research, but it also faces the problem of individual differences in human brain structure and function. In four independent ADHD datasets, Wang et al. (2017) applied several commonly used resting-state analysis, including regional homogeneity, amplitude of low-frequency fluctuation, and degree centrality, and found that there were no overlapping abnormal brain regions among datasets, although three of the four datasets were collected in the same site. In addition to the high heterogeneity of ADHD, due to the complexity and individual differences of the human brain, the co-registration process of fMRI data may also be one of the relevant factors. At present, the common method in fMRI researches (74% of 9,400 fMRI studies; Derrfuss and Mar, 2009) is to register the brains of different individuals on a unified Talairach or Montreal Neurological Institute template to define corresponding brain regions. Due to the lack of accurate information on the boundaries of brain regions and the large differences in the structure/function of brain regions among individuals, the results largely depend on the algorithm of the registration step (Zhang and Cootes, 2011).

Therefore, in the ADHD fMRI study, under the demand of multicenter data sharing, considering the individual brain differences, a method to define brain regions with fine-granularity and better structural/functional consistency across brains is warranted.

The Dense Individualized and Common Connectivity-based Cortical Landmarks (DICCCOL; Zhu et al., 2013) system defined 358 individualized landmarks with structural and functional consistency across different brains. The physiological basis of this method is the “connectional fingerprint concept” (Passingham et al., 2002), which premised that each brain’s cytoarchitectonic area has a unique set of extrinsic inputs and outputs that largely determines the functions that each brain area performs. This close relationship between structure and function had been supported by some evidence (Honey et al., 2009; Li et al., 2010; Zhu et al., 2012). Therefore, 358 DICCCOLs are 358 landmarks on the cortex with consistent similar fiber bundle patterns across individual subjects. Because of this good functional consistency across different brains, DICCCOL could be one of the most promising methods that may help us to observe ADHD without the interference of individual variability of cortical structure and function. For the same reason, DICCCOL also provides better comparability for results from different datasets. Another benefit of applying DICCCOL in researching psychiatric disorders is the potential usage for white-matter (WM) abnormity detection. ADHD was considered a psychiatric disorder with WM abnormity based on previous diffusion tensor imaging (DTI) research (van Ewijk et al., 2012; Aoki et al., 2018). Also, we have found some discrepant DICCCOLs in ADHD patients with different fiber bundle patterns (Wang et al., 2013).

Thus, in the current study, we adopted the DICCCOL system in two independent ADHD datasets. We hypothesized that children with ADHD (1) exhibit abnormal fiber bundle, (2) exhibit abnormal resting functional connectivities, and (3) exhibit consistent abnormalities across independent datasets.

## Materials and Methods

### Subjects

This study includes two independent datasets. These two datasets were collected for different purposes and using different scan parameters. Previous analysis of these datasets has been published elsewhere separately (An et al., 2013; Cao et al., 2013). Only diffusion-weighted MRI and RS-fMRI scans were used in the current study. The following inclusion criteria were used for the ADHD group: (a) age between 8 and 14 years at the time point of the scan; (b) diagnosis of ADHD by a clinician using the Schedule for Affective Disorders and Schizophrenia for School-Age Children–Present and Lifetime Version (Kaufman et al., 2000); and (c) right-handedness. The following exclusion criteria were used: (a) other Axis-I psychiatric diagnoses; (b) IQ < 80 using the Chinese Wechsler Intelligence Scale for Children (Gong and Cai, 1993); (c) psychotropic medication history; and (d) any significant medical or neurological conditions or a history of head injury. In the two datasets, the inclusion and exclusion criteria of the control group were basically the same as those of the ADHD group, but the subjects in the control group did not meet the diagnostic criteria of ADHD. There are 25 ADHD-combined-type children and 45 age- and sex-matched controls in dataset 1. For dataset 2, 11 ADHD-combined-type children and 26 controls who matched in age and sex are included. The demographic characteristics of all participants are shown in Table 1.

**TABLE 1:** Demographic characteristics of two datasets.

		ADHD	Control	*p*
Dataset 1 diffusion-weighted MRI	Number (m/f)	25 (24/1)	45 (35/10)	*p* = 0.09
	Age (years)	11.05 (1.68)	11.00 (1.40)	*p* = 0.89
	IQ	108.1 (16.7)	121.3 (13.6)	*p* < 0.001
	Inattention scores	27.0 (12.2)	16.1 (4.0)	*p* < 0.001
	Impulsivity scores	27.2 (11.1)	15.1 (4.0)	*p* < 0.001
Dataset 1 fMRI (6 participants were excluded for head motion)	Number (m/f)	23 (22/1)	41 (31/10)	*p* = 0.09
	Age (years)	11.08 (1.67)	11.06 (1.40)	*p* = 0.96
	IQ	108.65 (16.97)	122.27 (13.61)	*p* < 0.001
	Inattention scores	26.77 (4.00)	16.15 (3.87)	*p* < 0.001
	Impulsivity scores	25.36 (7.44)	15.27 (3.94)	*p* < 0.001
Dataset 2	Number (m/f)	11 (11/0)	26 (26/0)	
	Age (years)	11.71 (2.13)	11.98 (1.77)	*p* = 0.69
	IQ	114.36 (13.86)	119.04 (12.80)	*p* = 0.33
	Inattention scores	28.00 (4.38)	15.23 (2.54)	*p* < 0.001
	Impulsivity scores	25.55 (5.91)	13.04 (3.86)	*p* < 0.001

*Values are mean (standard deviation). Six subjects in Dataset 1 were excluded for head motion during fMRI preprocessing.*

The ADHD Rating Scale-IV forms were collected from the parents of ADHD children to assess the severity of ADHD symptoms. Written informed consent was obtained from each parent, and each child agreed to participate. The Institutional Review Board at the Health Center of Peking University approved this study.

### Imaging Parameters

Both two datasets with multimodal diffusion-weighted/RS-fMRI data are acquired in a SIEMENS TRIO 3T scanner at the State Key Laboratory of Cognitive Neuroscience and Learning, Beijing Normal University. The diffusion-weighted MRI images of dataset 1 are acquired using the following parameters: 49 axial slices, repetition time (TR) = 7,200 ms, echo time (TE) = 104 ms, flip angle = 90°, 64 diffusion directions, *b* = 1,000 s/mm2, thickness = 2.5 mm, slice spacing = 0 mm, and acquisition matrix = 128 × 128. As for dataset 2, the diffusion-weighted MRI images are acquired by following parameters: 47 axial slices, TR = 6,900 ms, TE = 104 ms, flip angle = 90°, 64 diffusion directions, *b* = 1,000 s/mm2, thickness = 2.5 mm, slice spacing = 2.5 mm, and acquisition matrix = 128 × 128. The RS-fMRI images of these two datasets shared similar parameters. They were acquired using an echo-planar imaging sequence with 33 axial slices, TR = 2,000 ms, TE = 30 ms, flip angle = 90°, slice thickness = 3 mm, slice spacing = 3.6 mm, in-plane resolution = 64 × 64, and 240 volumes.

### Image Preprocessing

Preprocessing of the diffusion-weighted MRI data include brain skull removal, head motion correction, and eddy current correction. Subsequently, fibers tracts, gray matter and WM tissue segmentations (Liu et al., 2007), and the cortical surface were generated based on the diffusion-weighted MRI data (Liu et al., 2008). Specifically, fiber tracking was performed via the MEDINRIA^1^. The fractional anisotropy threshold was 0.2, and the minimum fiber length was 20 mm. Brain tissue segmentation was conducted on the diffusion-weighted MRI data directly (Liu et al., 2007). Our DICCCOL landmark is identified based on WM fiber tracts and located on WM cortical surface derived from DTI data. So, we performed segmentation on DTI data directly instead of structural MRI to avoid any structural MRI-DTI registration error. Based on the WM tissue map, the cortical surface was reconstructed using the marching cubes algorithm (Liu et al., 2008). The RS-fMRI data preprocessing steps include motion correction, spatial smoothing, slice time correction, global drift removal, and bandpass filtering (0.01–0.1 Hz) via FSL FEAT software^2^. The DTI space was used as the standard space. RS-fMRI data were registered to the DTI space via the FSL FLIRT software (Jenkinson and Smith, 2001).

### Dense Individualized and Common Connectivity-Based Cortical Landmark Identification

We defined 358 DICCCOL ROIs (Zhu et al., 2013) based on diffusion-weighted MRI data on each participant’s brain. As mentioned earlier, this method has been proven to be reasonably consistent and reproducible in over 240 brains (Zhu et al., 2013). In brief, the DICCCOL prediction process is based on the consistency of WM fiber connection patterns across brains, and it is composed of three major steps: initial landmark selection, optimization of landmark locations, and determination of group-wise consistent DICCCOLs. Details of the process can be found in the previous publication (Zhu et al., 2013).

In the discussed steps, the shape of each fiber bundle will be quantified as a “trace-map.” In this way, the morphological differences between fiber bundles can be quantified as “trace-map distance.” The specific algorithm can be found in the previous publication (Li et al., 2010). According to our previous work (Wang et al., 2013), among ADHD patients, specific DICCCOL ROIs show a significantly higher trace-map distance to the given models compared with the normal controls. In the current study, using the same one-side *t*-test method (*p* < 0.05), abnormal DICCCOL ROIs in each dataset were identified. Then, we investigated whether there are overlapped results between the two datasets.

### Functional Connectivity Analysis

Because the DICCCOL method is based on the “Connectional Fingerprint Concept” (Passingham et al., 2002), which simply stated that only brain landmarks with similar WM fiber bundle morphology are considered to have functional correspondence among different individual brains and thus can be used as an ROI of functional analysis, in the current research, only DICCCOL ROIs without significant group differences in the trace-map distance were involved in resting-state functional connectivity analysis.

After the co-registration between fMRI and diffusion-weighted MRI data, we extracted the RS-fMRI BOLD signals of each DICCCOL ROI for each participant. Functional connectivities were calculated for each pair of DICCCOL ROIs. After Fisher’s r-to-z transform, we conducted between-group comparison (*p* < 0.05). Again, by overlapping the result of two datasets, we got a relatively consistent abnormal functional connectivity map.

### Correlation Analysis Between Functional Connectivity and Symptom Severity

To study the relationship between ADHD symptoms (ADHD-RS score) and abnormal functional connectivity with good cross-sample universality, we applied generalized linear regression, with covariances including age/sex/IQ.

## Results

### Demographic Data

The information for the demographics/clinical data of the participants is summarized in Table 1. For the analysis of continuous variables, a *t*-test was used to compare means. Categorical variables were compared with the chi-square test. The differences in sex and age were not significant.

### Discrepant Dense Individualized and Common Connectivity-Based Cortical Landmarks in Attention Deficit Hyperactivity Disorder

**FIGURE 1:**
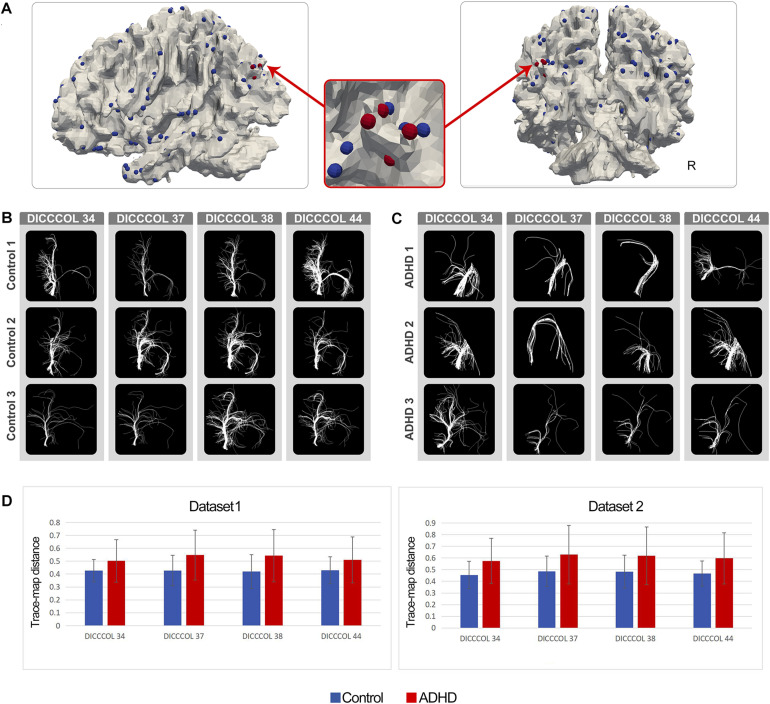
**(A)** Distribution of DICCCOLs: 34, 37, 38, and 44 (four red bubbles); **(B)** Fiber bundle of DICCCOLs 34, 37, 38, and 44 in three random NC brains; each row represents a participant; **(C)** Fiber bundle of DICCCOLs 34, 37, 38, and 44 in three random ADHD patients’ brains; each row represents a participant; **(D)** Trace-map distance of DICCCOLs 34, 37, 38, and 44 in dataset 1 and dataset 2.

As expected, in the ADHD group, certain DICCCOL ROIs displayed notably higher trace-map distance compared with the control group, which indicated that the fiber bundle patterns at these locations have higher variability. Four DICCCOL ROIs (DICCCOL numbers: 34, 37, 38, and 44) among them were shared by both datasets. The positions and trace map distance of these four DICCCOL ROIs, as well as the shape of the fiber bundles, are shown in Figure 1. The information of all 358 DICCCOL fiber bundle patterns and comparison between groups can be found in the Supplementary Materials.

### Results of Functional Connectivity Analysis

**FIGURE 2:**
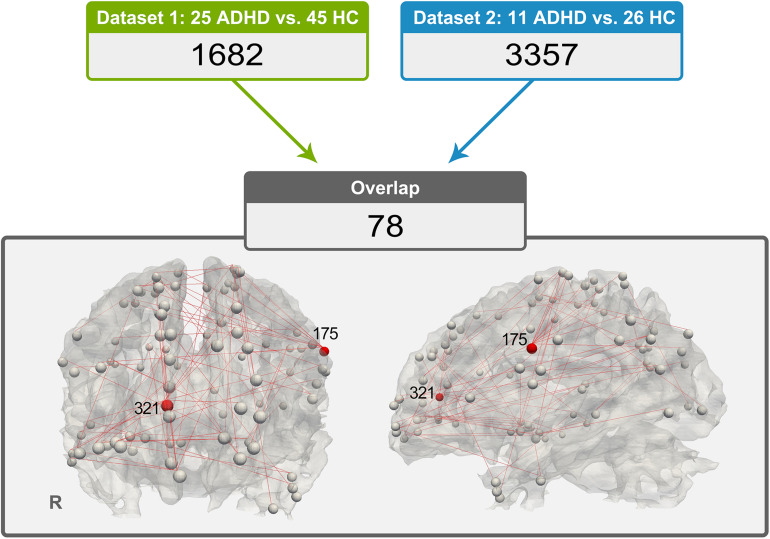
Distribution of 78 pairs of discrepant functional connectivity, which could indicate the importance of DICCCOL nos. 321 and 175 in this abnormal network (a relatively large number of red lines are connected).

The 78 consistent abnormal functional connectivities in the ADHD group across datasets are shown in Figure 2. Among the abnormal functional connectivities, 10 were related to DICCCOL 175, and nine were related to DICCCOL 321.

### Results of Correlation Analysis Between Functional Connectivity and Symptom Severity

**FIGURE 3:**
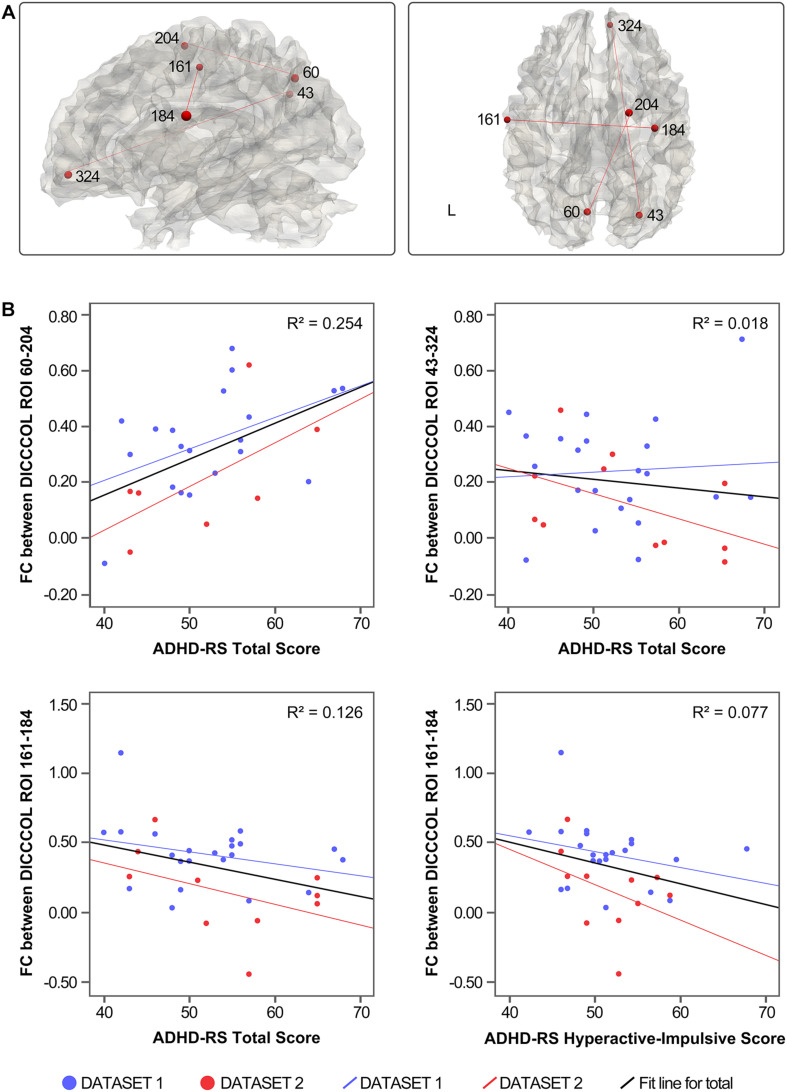
**(A)** Distribution of functional connectivities. **(B)** Correlation between functional connectivities and symptom severity.

Three of the 78 overlapping functional connectivities (Z score) significantly predicted the ADHD parent rating scale score. The result is shown in Figure 3. Based on the previous study that labeled DICCCOLs with functional meaning (Yuan et al., 2013), the physiological significance of these three functional connectivities is shown in Table 2.

**TABLE 2:** Physiological significance of functional connectivity related to the severity of ADHD symptoms.

DICCCOL number	Location	Brodmann area	Function
60	Left precuneus	7	None
∼204	Right superior frontal gyrus	6	Cognition, Language, Memory.
43	Right precuneus	19	Cognition, Attention, Language
∼324	Right anterior cingulate	10	None
161	Right precentral gyrus	4	Action, Attention, Memory, Emotion
∼184	Left precentral gyrus	43	Action, Language, Emotion, Language

## Discussion

In the current study, after the DICCCOL prediction procedure, we found that ADHD patients showed notably discrepant fiber bundle patterns on a number of DICCCOLs, and four of them were shared by both datasets. As shown in Figure 1, DICCCOLs 34, 37, 38, and 44 were located near the left parietooccipital junction and had a very close spatial position. In addition, 78 abnormal functional connectivities exhibited good cross-sample consistency. Three of them were significantly related to the severity of ADHD symptoms.

The DICCCOL method established 358 individual landmarks on each participant’s brain. DICCCOLs with the same number were considered to have good functional correspondence among different participants. A meta-analysis labeled each DICCCOL with functional meaning by registering them to 1,110 previous task-fMRI research (Yuan et al., 2013). The result interpretation of the current study is based on this publication.

We found that DICCCOL nos. 34, 37, 38, and 44, located near the junction of the left parietal and occipital lobe, exhibited abnormal fiber bundle structure in the ADHD group. These four DICCCOLs were very close in spatial location, and there were no other DICCCOLs between them. Therefore, we speculated that these four DICCCOLs might be small subregions of a larger functional brain region. These abnormalities of the four adjacent landmarks may indicate the structural and, thus, functional (Passingham et al., 2002) abnormalities in this larger brain region. Furthermore, among the 358 landmarks all over the brain, only four adjacent ones of them were found, which also highlighted the significance of this large brain area in the pathological mechanism of ADHD. According to previous meta-analysis (Yuan et al., 2013), the functions of these four DICCCOLs are undefined. DICCCOL nos. 34, 37, 38, and 44 were located in the left posterior parietal lobe/upper occipital lobe, and its spatial distribution was similar to the DMN components. The most consistent components of the DMN are medial regions (medial prefrontal, posterior cingulate/precuneus) and lateral regions (posterior parietal lobe) of the brain. These structures are usually reduced in activity in cognitive tasks requiring attention (Raichle et al., 2001; Raichle, 2014). Therefore, it can also explain that these four DICCCOLs were not activated in as much as 1,110 previous studies and thus were labeled as undefined (Yuan et al., 2013). So, the results of the current study supported the hypothesis that ADHD patients have abnormalities in posterior DMN.

By applying functional connectivity analysis between DICCCOL ROIs with no difference in WM fiber bundle morphology, we obtained 1,682 functional connectivities with between-group differences in dataset 1 and 3,357 in dataset 2. Seventy-eight of them exhibited good consistency across datasets. Interestingly, we found that a quarter (19 of 78) of these abnormal functional connectivities were associated with DICCCOL ROI nos. 175 and 321. Therefore, these two DICCCOLs with abnormal functional connectivities, which are consistently shown in two separate datasets, may play an important role in the pathogenesis of ADHD.

According to the previous meta-analysis (Yuan et al., 2013), DICCCOL 175 was located in the left precentral gyrus and was related to “Cognition. Speech. Language. Working Memory. Attention.” Functional and structural abnormalities of the precentral gyrus are common in ADHD studies. In a recent resting-state functional connectivity study of ADHD, Guo also used whole-brain functional connectivity to classify features for machine learning and found that two of the four features with the best discriminability were related to the precentral gyrus, namely the precentral gyrus–prefrontal lobe and the precentral gyrus–superior temporal gyrus (Guo et al., 2020). In a young adult with ADHD, Krista found that the cerebral cortex thickness of the precentral and postcentral gyrus was the only brain regions associated with the persistence of ADHD, and the cluster in the precentral gyrus was close to that of DICCCOL 175 (Lisdahl et al., 2016). Bernis found that the cortical of the bilateral precentral gyrus and supplementary motor area in ADHD children was thicker than that in normal controls, and the thickness was positively correlated with the severity of ADHD symptoms (Sutcubasi Kaya et al., 2018). Therefore, in the current study, the abnormal functional connectivity of the 175 DICCCOL ROI supported the findings in previous researches. Considering that ADHD children are asked to “keep still” during a scan, for individuals with more prominent hyperactivity symptoms, it is necessary to suppress motor-related impulses. This finding may reflect the inhibition of motor impulse in ADHD children under a resting state.

DICCCOL 321 located in the right anterior cingulate gyrus is related to cognition and emotion (Yuan et al., 2013). During the various task, such as go/no go, response inhibition, attention, and hypoactivity of ACC were found by fMRI, PET, and event-related potential in ADHD patients (Rubia et al., 1999; Durston et al., 2003, 2007). A meta-analysis that included 16 task state functional brain image studies also found that ACC was one of the brain regions with consistent hypoactivity in ADHD patients across studies (Dickstein et al., 2006). In addition, after the DMN interference hypothesis was put forward (Kelly et al., 2008), ACC has been widely concerned because of its weak negative correlation with DMN components in ADHD children and adults (Sato et al., 2012; Sun et al., 2012). Furthermore, the efficacy of stimulant treatment in patients with ADHD-combined type is related to the volume of right ACC (Semrud-Clikeman et al., 2014). In ADHD adults, using single-voxel proton magnetic resonance spectroscopy, glutamate levels in the ACC were higher than the control group and positively correlated with ADHD symptomatology (Bauer et al., 2016). Therefore, the abnormal ACC functional connectivities found in this study are consistent with previous studies, reflecting the abnormal brain activity outside the default network of ADHD patients under resting state.

The functional connectivities related to ADHD symptoms were mostly between the default network components and other brain networks, such as the right precuneus and the right ACC (DICCCOL 43–324) and left precuneus and right superior frontal gyrus (DICCCOL 60–204), which reflected the abnormal function of DMN and other networks in resting state. In addition, functional connectivity between the bilateral precentral gyrus (DICCCOL 161–184) was associated with hyperactivity impulse scores. Together with the discussed findings of abnormal functional connectivity in the precentral gyrus (DICCCOL 175), this finding highlights the significance of abnormal activity of the precentral gyrus in patients with ADHD under resting state. In contrast, there is no functional connectivities related to the inattention score of ADHD-RS, which may be related to the low demand on attention level in the resting state. This issue could be verified in the future by combining task and resting-state scans.

The current study has the following limitations: (1) we abandoned some abnormal DICCCOLs during fMRI analysis, so 322 DICCCOLs are not a perfect representation of the “whole-brain;” however, the brain activity with more ADHD characteristics may be reflected in these DICCCOLs. As mentioned in *Method*, because these abnormal DICCCOLs do not have good WM fiber bundle morphological similarity, we cannot arbitrarily determine their functional correspondence. This is the temporary limitation of the DICCCOL method and a critical issue to be overcome in the application of future disease research. (2) The “connectional fingerprint concept” (Passingham et al., 2002) is a theory based on healthy individuals, so its application in the disease population needs to be cautious. (3) Small sample size may influence the results; also, the imbalance of sample size will lead to lower statistical efficiency. The findings of the current study need to be validated on a larger multicenter sample.

In this study, we used the DICCCOL method and replicated some findings consistent with previous studies on two independent ADHD samples, and some unique findings were also found: there were morphological abnormalities in the WM tracts in the left posterior cortex of the DMN in children with ADHD; in the resting state, the abnormal functional connectivities with good cross-sample consistency mainly involved attention, motion, emotion, and working memory (DICCCOL 175/321). This study provides a new possibility for multicenter and large sample ADHD study in the future. Although the physiological significance of the part of the DICCCOL landmarks is still unclear, Castellanos points out that effective biomarkers do not necessarily have a clear neurophysiological significance or explanation (Castellanos et al., 2013). From this point of view, DICCCOL, which is easy to operate and has good interindividual functional consistency, is a possible direction for the clinical application of resting-state fMRI.

## Data Availability Statement

The raw data supporting the conclusions of this article will be made available by the authors, without undue reservation.

## Ethics Statement

The studies involving human participants were reviewed and approved by the Institutional Review Board at the Health Center of Peking University. Written informed consent to participate in this study was provided by the participants’ legal guardian/next of kin.

## Author Contributions

YW, PW, and SZ: conception and design. PW, XJ, HC, SZ, XL, BY, and YW: analysis and interpretation. PW, QC, LS, and LL: data collection. PW and SZ writing the manuscript. XJ and QC: critical revision of the manuscript. PW, SZ, and XJ: statistical analysis. YW: overall responsibility. All authors contributed to the article and approved the submitted version.

## Conflict of Interest

The authors declare that the research was conducted in the absence of any commercial or financial relationships that could be construed as a potential conflict of interest.

## Footnotes

1 http://www-sop.inria.fr/asclepios/software/MedINRIA/

2 http://fsl.fmrib.ox.ac.uk/fsl/feat5/
