# Supplementary material for: Assessing Fine-Granularity Structural and Functional Connectivity in Children With Attention Deficit Hyperactivity Disorder
Source: Front Hum Neurosci. 2020 Nov 13;14:594830. doi: 10.3389/fnhum.2020.594830 (PMC7691597; doi:10.3389/fnhum.2020.594830)
Supplement: Supplementary file 1 [file Table_1.DOCX]

Attention deficit hyperactivity disorder (ADHD) is one of the most common neurobehavioral psychiatric disorders of childhood with high prevalence rates (5.47%). The typical symptoms are characterized as excessive inattention, hyperactivity/impulsiveness or their combinations. The pathological mechanism of ADHD is still unclear. High heterogeneity in ADHD studies has become an important issue. In addition to the complex etiology, individual differences in brain structure and function are also important factors. This problem is especially prominent in multi-center/large sample studies.

In the current study, we applied the DICCCOL method on two independent datasets. The DICCCOL method established 358 individual landmarks on each participant's brain according to morphology of white matter fiber bundle. DICCCOL landmarks with the same number were considered to have good functional correspondence among different participants.

There were several findings with good cross sample consistency: there were morphological abnormalities in the white matter tracts in the left posterior cortex of the DMN in children with ADHD; in the resting state, the abnormal functional connectivities with good cross sample consistency mainly involved attention, motion, emotion, and working memory (DICCCOL 175/321). This study provides a new possibility for multi-center and large sample ADHD study in the future.
