# Supplementary material for: Assessing Fine-Granularity Structural and Functional Connectivity in Children With Attention Deficit Hyperactivity Disorder
Source: Front Hum Neurosci. 2020 Nov 13;14:594830. doi: 10.3389/fnhum.2020.594830 (PMC7691597; doi:10.3389/fnhum.2020.594830)
Supplement: Supplementary file 2 [file Table_2.docx]

**Supplementary Material for Review**

Supplementary Table. The information of 358 DICCCOL fiber bundle patterns and comparison between groups.

| DICCCOL number | Dataset 1 Control | Dataset 1 ADHD | *p* | Dataset 2 Control | Dataset 2 ADHD | *p* |
| --- | --- | --- | --- | --- | --- | --- |
| 1 | 0.51±0.10 | 0.54±0.13 | 0.329 | 0.49±0.10 | 0.46±0.07 | 0.367 |
| 2 | 0.55±0.11 | 0.55±0.11 | 0.894 | 0.51±0.13 | 0.54±0.13 | 0.526 |
| 3 | 0.51±0.11 | 0.54±0.14 | 0.279 | 0.48±0.09 | 0.48±0.12 | 0.949 |
| 4 | 0.45±0.10 | 0.46±0.13 | 0.866 | 0.48±0.13 | 0.45±0.10 | 0.547 |
| 5 | 0.51±0.14 | 0.49±0.10 | 0.512 | 0.48±0.12 | 0.43±0.08 | 0.270 |
| 6 | 0.55±0.12 | 0.51±0.10 | 0.114 | 0.54±0.13 | 0.66±0.19 | 0.041^*^ |
| 7 | 0.47±0.09 | 0.49±0.10 | 0.459 | 0.49±0.09 | 0.49±0.11 | 0.832 |
| 8 | 0.46±0.09 | 0.49±0.13 | 0.243 | 0.47±0.11 | 0.49±0.14 | 0.624 |
| 9 | 0.52±0.10 | 0.53±0.10 | 0.838 | 0.49±0.09 | 0.52±0.10 | 0.398 |
| 10 | 0.45±0.08 | 0.44±0.09 | 0.557 | 0.44±0.09 | 0.46±0.07 | 0.468 |
| 11 | 0.44±0.10 | 0.49±0.14 | 0.059 | 0.45±0.13 | 0.45±0.07 | 0.969 |
| 12 | 0.43±0.08 | 0.46±0.13 | 0.290 | 0.43±0.07 | 0.45±0.08 | 0.366 |
| 13 | 0.43±0.08 | 0.46±0.12 | 0.197 | 0.42±0.05 | 0.41±0.09 | 0.783 |
| 14 | 0.45±0.06 | 0.44±0.07 | 0.207 | 0.43±0.08 | 0.43±0.08 | 0.992 |
| 15 | 0.52±0.12 | 0.53±0.12 | 0.697 | 0.53±0.12 | 0.53±0.09 | 0.948 |
| 16 | 0.49±0.09 | 0.48±0.09 | 0.618 | 0.47±0.09 | 0.52±0.11 | 0.109 |
| 17 | 0.51±0.12 | 0.55±0.13 | 0.181 | 0.52±0.16 | 0.63±0.11 | 0.043^*^ |
| 18 | 0.45±0.08 | 0.48±0.12 | 0.135 | 0.45±0.06 | 0.46±0.08 | 0.693 |
| 19 | 0.45±0.09 | 0.44±0.09 | 0.644 | 0.42±0.08 | 0.44±0.06 | 0.523 |
| 20 | 0.48±0.12 | 0.49±0.13 | 0.879 | 0.48±0.10 | 0.50±0.11 | 0.532 |
| 21 | 0.46±0.09 | 0.48±0.14 | 0.291 | 0.41±0.08 | 0.46±0.10 | 0.200 |
| 22 | 0.44±0.09 | 0.50±0.13 | 0.015^*^ | 0.46±0.09 | 0.42±0.09 | 0.334 |
| 23 | 0.43±0.10 | 0.43±0.12 | 0.797 | 0.40±0.08 | 0.43±0.08 | 0.341 |
| 24 | 0.64±0.15 | 0.59±0.18 | 0.184 | 0.64±0.13 | 0.72±0.27 | 0.222 |
| 25 | 0.52±0.11 | 0.59±0.15 | 0.020^*^ | 0.56±0.14 | 0.61±0.10 | 0.309 |
| 26 | 0.45±0.11 | 0.48±0.11 | 0.341 | 0.46±0.08 | 0.45±0.06 | 0.943 |
| 27 | 0.41±0.10 | 0.44±0.14 | 0.323 | 0.41±0.11 | 0.42±0.12 | 0.838 |
| 28 | 0.42±0.10 | 0.45±0.11 | 0.202 | 0.42±0.12 | 0.43±0.08 | 0.872 |
| 29 | 0.69±0.18 | 0.67±0.17 | 0.612 | 0.64±0.15 | 0.74±0.13 | 0.076 |
| 30 | 0.37±0.08 | 0.40±0.08 | 0.162 | 0.38±0.10 | 0.41±0.09 | 0.505 |
| 31 | 0.44±0.10 | 0.45±0.07 | 0.486 | 0.44±0.10 | 0.45±0.06 | 0.624 |
| 32 | 0.53±0.13 | 0.50±0.09 | 0.243 | 0.55±0.11 | 0.51±0.13 | 0.303 |
| 33 | 0.43±0.10 | 0.49±0.10 | 0.025^*^ | 0.42±0.10 | 0.45±0.09 | 0.392 |
| 34 | 0.43±0.09 | 0.50±0.17 | 0.012^*^ | 0.45±0.12 | 0.58±0.19 | 0.024^*^ |
| 35 | 0.52±0.10 | 0.53±0.07 | 0.668 | 0.51±0.10 | 0.53±0.11 | 0.622 |
| 36 | 0.48±0.10 | 0.51±0.09 | 0.333 | 0.47±0.10 | 0.49±0.07 | 0.470 |
| 37 | 0.43±0.12 | 0.55±0.19 | 0.001^**^ | 0.49±0.13 | 0.63±0.25 | 0.028^*^ |
| 38 | 0.42±0.13 | 0.54±0.20 | 0.002** | 0.48±0.14 | 0.62±0.25 | 0.040* |
| 39 | 0.63±0.10 | 0.60±0.11 | 0.182 | 0.58±0.11 | 0.63±0.12 | 0.220 |
| 40 | 0.45±0.11 | 0.46±0.13 | 0.667 | 0.44±0.09 | 0.44±0.11 | 0.967 |
| 41 | 0.41±0.09 | 0.44±0.08 | 0.143 | 0.41±0.07 | 0.44±0.06 | 0.139 |
| 42 | 0.42±0.07 | 0.48±0.12 | 0.011^*^ | 0.44±0.09 | 0.50±0.19 | 0.232 |
| 43 | 0.48±0.12 | 0.49±0.15 | 0.878 | 0.44±0.11 | 0.44±0.06 | 0.832 |
| 44 | 0.42±0.10 | 0.51±0.18 | 0.011^*^ | 0.47±0.11 | 0.60±0.22 | 0.019^*^ |
| 45 | 0.46±0.09 | 0.48±0.08 | 0.220 | 0.46±0.11 | 0.47±0.12 | 0.678 |
| 46 | 0.65±0.12 | 0.64±0.11 | 0.761 | 0.63±0.17 | 0.64±0.07 | 0.882 |
| 47 | 0.44±0.09 | 0.45±0.08 | 0.890 | 0.44±0.10 | 0.50±0.09 | 0.095 |
| 48 | 0.39±0.07 | 0.40±0.09 | 0.705 | 0.38±0.07 | 0.39±0.06 | 0.547 |
| 49 | 0.42±0.08 | 0.39±0.07 | 0.181 | 0.40±0.08 | 0.44±0.12 | 0.216 |
| 50 | 0.40±0.07 | 0.40±0.07 | 0.995 | 0.39±0.07 | 0.44±0.09 | 0.044^*^ |
| 51 | 0.39±0.07 | 0.39±0.07 | 0.955 | 0.39±0.08 | 0.37±0.06 | 0.552 |
| 52 | 0.69±0.16 | 0.67±0.16 | 0.535 | 0.65±0.21 | 0.72±0.16 | 0.312 |
| 53 | 0.41±0.06 | 0.42±0.07 | 0.724 | 0.41±0.06 | 0.39±0.07 | 0.527 |
| 54 | 0.84±0.16 | 0.84±0.15 | 0.930 | 0.82±0.12 | 0.77±0.16 | 0.313 |
| 55 | 0.51±0.12 | 0.51±0.11 | 0.981 | 0.49±0.12 | 0.51±0.12 | 0.549 |
| 56 | 0.41±0.09 | 0.46±0.11 | 0.044^*^ | 0.41±0.09 | 0.39±0.05 | 0.388 |
| 57 | 0.55±0.10 | 0.59±0.17 | 0.271 | 0.55±0.12 | 0.58±0.13 | 0.475 |
| 58 | 0.42±0.09 | 0.42±0.07 | 0.768 | 0.45±0.11 | 0.41±0.12 | 0.291 |
| 59 | 0.86±0.13 | 0.91±0.14 | 0.115 | 0.84±0.11 | 0.84±0.23 | 0.909 |
| 60 | 0.49±0.08 | 0.51±0.08 | 0.428 | 0.49±0.09 | 0.48±0.07 | 0.752 |
| 61 | 0.40±0.09 | 0.39±0.08 | 0.484 | 0.38±0.08 | 0.38±0.05 | 0.905 |
| 62 | 0.38±0.08 | 0.38±0.07 | 0.888 | 0.36±0.06 | 0.39±0.08 | 0.229 |
| 63 | 0.39±0.09 | 0.38±0.11 | 0.500 | 0.38±0.09 | 0.38±0.07 | 0.930 |
| 64 | 0.52±0.09 | 0.51±0.09 | 0.683 | 0.51±0.13 | 0.49±0.10 | 0.726 |
| 65 | 0.38±0.05 | 0.40±0.06 | 0.094 | 0.37±0.07 | 0.38±0.04 | 0.693 |
| 66 | 0.62±0.14 | 0.68±0.20 | 0.117 | 0.59±0.15 | 0.60±0.16 | 0.932 |
| 67 | 0.41±0.06 | 0.43±0.08 | 0.337 | 0.41±0.06 | 0.41±0.05 | 0.799 |
| 68 | 0.38±0.08 | 0.38±0.08 | 0.634 | 0.37±0.07 | 0.38±0.10 | 0.803 |
| 69 | 0.63±0.12 | 0.69±0.18 | 0.064 | 0.60±0.15 | 0.61±0.17 | 0.923 |
| 70 | 0.43±0.07 | 0.46±0.08 | 0.191 | 0.42±0.08 | 0.42±0.05 | 0.960 |
| 71 | 0.68±0.14 | 0.73±0.18 | 0.184 | 0.66±0.20 | 0.68±0.17 | 0.752 |
| 72 | 0.36±0.09 | 0.37±0.07 | 0.560 | 0.37±0.12 | 0.37±0.06 | 0.975 |
| 73 | 0.64±0.13 | 0.67±0.16 | 0.437 | 0.61±0.14 | 0.63±0.10 | 0.742 |
| 74 | 0.65±0.11 | 0.64±0.15 | 0.793 | 0.59±0.11 | 0.68±0.12 | 0.030^*^ |
| 75 | 0.54±0.09 | 0.52±0.11 | 0.390 | 0.50±0.08 | 0.55±0.10 | 0.117 |
| 76 | 0.59±0.12 | 0.56±0.11 | 0.212 | 0.53±0.13 | 0.62±0.12 | 0.054 |
| 77 | 0.47±0.12 | 0.45±0.10 | 0.579 | 0.41±0.09 | 0.43±0.06 | 0.438 |
| 78 | 0.41±0.09 | 0.43±0.07 | 0.445 | 0.41±0.10 | 0.41±0.08 | 0.931 |
| 79 | 0.38±0.07 | 0.39±0.05 | 0.728 | 0.39±0.05 | 0.36±0.07 | 0.169 |
| 80 | 0.44±0.06 | 0.45±0.08 | 0.571 | 0.43±0.06 | 0.47±0.21 | 0.464 |
| 81 | 0.34±0.08 | 0.38±0.09 | 0.081 | 0.35±0.07 | 0.32±0.05 | 0.187 |
| 82 | 0.32±0.06 | 0.34±0.09 | 0.437 | 0.32±0.06 | 0.33±0.08 | 0.708 |
| 83 | 0.35±0.08 | 0.36±0.07 | 0.591 | 0.36±0.06 | 0.36±0.08 | 0.942 |
| 84 | 0.35±0.09 | 0.37±0.07 | 0.473 | 0.36±0.10 | 0.35±0.06 | 0.783 |
| 85 | 0.31±0.07 | 0.33±0.09 | 0.286 | 0.32±0.07 | 0.31±0.05 | 0.860 |
| 86 | 0.34±0.09 | 0.36±0.10 | 0.532 | 0.33±0.08 | 0.34±0.07 | 0.757 |
| 87 | 0.65±0.11 | 0.68±0.18 | 0.354 | 0.63±0.13 | 0.67±0.17 | 0.436 |
| 88 | 0.57±0.14 | 0.55±0.13 | 0.443 | 0.53±0.16 | 0.51±0.09 | 0.675 |
| 89 | 0.32±0.07 | 0.35±0.08 | 0.148 | 0.34±0.07 | 0.32±0.06 | 0.384 |
| 90 | 0.38±0.07 | 0.39±0.07 | 0.422 | 0.38±0.05 | 0.37±0.08 | 0.679 |
| 91 | 0.30±0.08 | 0.33±0.09 | 0.130 | 0.31±0.07 | 0.30±0.04 | 0.631 |
| 92 | 0.58±0.13 | 0.60±0.12 | 0.586 | 0.58±0.14 | 0.59±0.12 | 0.881 |
| 93 | 0.39±0.08 | 0.41±0.05 | 0.182 | 0.40±0.10 | 0.39±0.05 | 0.833 |
| 94 | 0.52±0.12 | 0.49±0.11 | 0.278 | 0.52±0.15 | 0.45±0.07 | 0.182 |
| 95 | 0.44±0.07 | 0.47±0.07 | 0.064 | 0.46±0.08 | 0.46±0.07 | 0.956 |
| 96 | 0.35±0.08 | 0.35±0.07 | 0.817 | 0.35±0.07 | 0.34±0.05 | 0.607 |
| 97 | 0.57±0.12 | 0.60±0.11 | 0.354 | 0.58±0.11 | 0.53±0.11 | 0.160 |
| 98 | 0.58±0.13 | 0.62±0.11 | 0.200 | 0.56±0.13 | 0.59±0.07 | 0.465 |
| 99 | 0.41±0.07 | 0.43±0.09 | 0.349 | 0.42±0.08 | 0.44±0.07 | 0.437 |
| 100 | 0.54±0.12 | 0.58±0.08 | 0.140 | 0.53±0.13 | 0.50±0.09 | 0.472 |
| 101 | 0.64±0.12 | 0.70±0.14 | 0.076 | 0.62±0.16 | 0.68±0.19 | 0.352 |
| 102 | 0.37±0.06 | 0.40±0.05 | 0.033^*^ | 0.37±0.06 | 0.38±0.05 | 0.440 |
| 103 | 0.35±0.07 | 0.36±0.07 | 0.552 | 0.33±0.05 | 0.37±0.07 | 0.104 |
| 104 | 0.29±0.08 | 0.29±0.08 | 0.706 | 0.30±0.06 | 0.30±0.07 | 0.815 |
| 105 | 0.39±0.05 | 0.39±0.07 | 0.883 | 0.40±0.05 | 0.37±0.05 | 0.179 |
| 106 | 0.40±0.06 | 0.43±0.08 | 0.095 | 0.41±0.07 | 0.39±0.08 | 0.284 |
| 107 | 0.60±0.12 | 0.62±0.11 | 0.456 | 0.56±0.10 | 0.65±0.11 | 0.027^*^ |
| 108 | 0.69±0.09 | 0.68±0.10 | 0.765 | 0.61±0.08 | 0.65±0.11 | 0.140 |
| 109 | 0.52±0.08 | 0.51±0.14 | 0.951 | 0.50±0.10 | 0.54±0.09 | 0.255 |
| 110 | 0.37±0.07 | 0.39±0.08 | 0.386 | 0.37±0.08 | 0.36±0.06 | 0.925 |
| 111 | 0.37±0.08 | 0.38±0.07 | 0.548 | 0.38±0.09 | 0.39±0.10 | 0.960 |
| 112 | 0.31±0.08 | 0.30±0.08 | 0.700 | 0.32±0.06 | 0.30±0.07 | 0.303 |
| 113 | 0.64±0.12 | 0.66±0.16 | 0.486 | 0.64±0.14 | 0.64±0.13 | 0.996 |
| 114 | 0.54±0.10 | 0.57±0.08 | 0.312 | 0.54±0.11 | 0.54±0.07 | 0.857 |
| 115 | 0.41±0.06 | 0.42±0.07 | 0.448 | 0.40±0.09 | 0.45±0.03 | 0.087 |
| 116 | 0.51±0.12 | 0.49±0.10 | 0.487 | 0.47±0.12 | 0.50±0.05 | 0.400 |
| 117 | 0.45±0.08 | 0.43±0.10 | 0.415 | 0.44±0.11 | 0.44±0.13 | 0.834 |
| 118 | 0.37±0.07 | 0.39±0.07 | 0.391 | 0.38±0.07 | 0.34±0.05 | 0.061 |
| 119 | 0.29±0.05 | 0.29±0.05 | 0.974 | 0.31±0.12 | 0.28±0.09 | 0.443 |
| 120 | 0.28±0.06 | 0.28±0.04 | 0.836 | 0.28±0.05 | 0.28±0.06 | 0.888 |
| 121 | 0.33±0.05 | 0.34±0.05 | 0.743 | 0.34±0.08 | 0.33±0.06 | 0.600 |
| 122 | 0.52±0.09 | 0.50±0.08 | 0.358 | 0.49±0.10 | 0.53±0.07 | 0.210 |
| 123 | 0.50±0.11 | 0.47±0.09 | 0.176 | 0.50±0.13 | 0.50±0.07 | 0.939 |
| 124 | 0.55±0.09 | 0.60±0.12 | 0.049^*^ | 0.56±0.18 | 0.52±0.10 | 0.510 |
| 125 | 0.57±0.15 | 0.63±0.18 | 0.116 | 0.50±0.15 | 0.50±0.12 | 0.913 |
| 126 | 0.36±0.07 | 0.36±0.08 | 0.848 | 0.35±0.11 | 0.37±0.11 | 0.662 |
| 127 | 0.55±0.11 | 0.53±0.08 | 0.380 | 0.54±0.10 | 0.55±0.10 | 0.781 |
| 128 | 0.44±0.09 | 0.44±0.10 | 0.925 | 0.46±0.10 | 0.45±0.11 | 0.678 |
| 129 | 0.32±0.07 | 0.31±0.06 | 0.649 | 0.30±0.06 | 0.31±0.13 | 0.646 |
| 130 | 0.29±0.05 | 0.32±0.06 | 0.035^*^ | 0.30±0.06 | 0.28±0.05 | 0.179 |
| 131 | 0.24±0.07 | 0.23±0.06 | 0.250 | 0.22±0.06 | 0.25±0.07 | 0.215 |
| 132 | 0.31±0.07 | 0.31±0.08 | 0.740 | 0.33±0.09 | 0.35±0.11 | 0.569 |
| 133 | 0.22±0.06 | 0.22±0.08 | 0.964 | 0.21±0.06 | 0.23±0.07 | 0.475 |
| 134 | 0.32±0.08 | 0.28±0.07 | 0.060 | 0.29±0.06 | 0.34±0.10 | 0.046^*^ |
| 135 | 0.34±0.07 | 0.37±0.10 | 0.101 | 0.36±0.08 | 0.34±0.07 | 0.487 |
| 136 | 0.55±0.11 | 0.55±0.11 | 0.879 | 0.49±0.09 | 0.58±0.13 | 0.020^*^ |
| 137 | 0.32±0.05 | 0.32±0.04 | 0.694 | 0.34±0.07 | 0.31±0.05 | 0.220 |
| 138 | 0.54±0.17 | 0.55±0.17 | 0.813 | 0.46±0.17 | 0.44±0.11 | 0.766 |
| 139 | 0.53±0.16 | 0.55±0.17 | 0.592 | 0.46±0.16 | 0.45±0.08 | 0.830 |
| 140 | 0.55±0.11 | 0.58±0.10 | 0.289 | 0.58±0.13 | 0.49±0.10 | 0.067 |
| 141 | 0.23±0.05 | 0.24±0.05 | 0.287 | 0.23±0.04 | 0.23±0.05 | 0.659 |
| 142 | 0.64±0.13 | 0.64±0.15 | 0.924 | 0.59±0.14 | 0.66±0.16 | 0.201 |
| 143 | 0.47±0.10 | 0.50±0.09 | 0.252 | 0.49±0.10 | 0.54±0.11 | 0.130 |
| 144 | 0.35±0.10 | 0.37±0.11 | 0.448 | 0.37±0.11 | 0.37±0.08 | 0.964 |
| 145 | 0.53±0.09 | 0.52±0.08 | 0.637 | 0.53±0.11 | 0.55±0.13 | 0.639 |
| 146 | 0.56±0.12 | 0.59±0.13 | 0.394 | 0.56±0.12 | 0.61±0.09 | 0.189 |
| 147 | 0.46±0.11 | 0.44±0.13 | 0.411 | 0.46±0.14 | 0.43±0.20 | 0.676 |
| 148 | 0.55±0.11 | 0.57±0.11 | 0.453 | 0.54±0.09 | 0.59±0.09 | 0.138 |
| 149 | 0.23±0.08 | 0.22±0.10 | 0.667 | 0.22±0.08 | 0.22±0.06 | 0.979 |
| 150 | 0.72±0.11 | 0.72±0.12 | 0.872 | 0.71±0.13 | 0.65±0.09 | 0.195 |
| 151 | 0.51±0.15 | 0.50±0.14 | 0.845 | 0.48±0.12 | 0.48±0.13 | 0.968 |
| 152 | 0.23±0.07 | 0.24±0.12 | 0.850 | 0.23±0.07 | 0.23±0.06 | 0.826 |
| 153 | 0.72±0.11 | 0.68±0.11 | 0.094 | 0.66±0.12 | 0.74±0.13 | 0.088 |
| 154 | 0.50±0.10 | 0.50±0.09 | 0.973 | 0.48±0.12 | 0.48±0.08 | 0.836 |
| 155 | 0.50±0.14 | 0.47±0.14 | 0.335 | 0.48±0.16 | 0.46±0.15 | 0.650 |
| 156 | 0.49±0.12 | 0.51±0.23 | 0.706 | 0.49±0.12 | 0.48±0.12 | 0.893 |
| 157 | 0.51±0.10 | 0.54±0.08 | 0.231 | 0.50±0.11 | 0.53±0.10 | 0.536 |
| 158 | 0.65±0.09 | 0.63±0.09 | 0.551 | 0.63±0.07 | 0.67±0.09 | 0.101 |
| 159 | 0.51±0.10 | 0.51±0.08 | 0.979 | 0.48±0.10 | 0.49±0.04 | 0.730 |
| 160 | 0.27±0.06 | 0.28±0.09 | 0.389 | 0.28±0.09 | 0.25±0.03 | 0.265 |
| 161 | 0.36±0.08 | 0.35±0.09 | 0.859 | 0.35±0.09 | 0.37±0.07 | 0.449 |
| 162 | 0.36±0.07 | 0.35±0.06 | 0.446 | 0.35±0.11 | 0.38±0.08 | 0.415 |
| 163 | 0.48±0.09 | 0.53±0.17 | 0.116 | 0.52±0.14 | 0.46±0.10 | 0.251 |
| 164 | 0.40±0.07 | 0.42±0.08 | 0.395 | 0.42±0.08 | 0.41±0.08 | 0.824 |
| 165 | 0.51±0.12 | 0.51±0.12 | 0.900 | 0.48±0.12 | 0.50±0.04 | 0.635 |
| 166 | 0.67±0.16 | 0.67±0.17 | 0.912 | 0.61±0.14 | 0.67±0.19 | 0.313 |
| 167 | 0.42±0.07 | 0.42±0.05 | 0.938 | 0.40±0.07 | 0.43±0.08 | 0.263 |
| 168 | 0.55±0.15 | 0.57±0.09 | 0.687 | 0.48±0.13 | 0.48±0.12 | 0.972 |
| 169 | 0.24±0.05 | 0.27±0.05 | 0.053 | 0.25±0.05 | 0.23±0.06 | 0.465 |
| 170 | 0.46±0.08 | 0.52±0.07 | 0.003^**^ | 0.47±0.08 | 0.42±0.07 | 0.096 |
| 171 | 0.40±0.09 | 0.40±0.06 | 0.926 | 0.40±0.06 | 0.40±0.06 | 0.953 |
| 172 | 0.26±0.08 | 0.23±0.07 | 0.068 | 0.26±0.07 | 0.25±0.07 | 0.711 |
| 173 | 0.66±0.15 | 0.68±0.16 | 0.631 | 0.62±0.16 | 0.72±0.26 | 0.166 |
| 174 | 0.25±0.08 | 0.27±0.07 | 0.438 | 0.30±0.07 | 0.26±0.04 | 0.082 |
| 175 | 0.65±0.14 | 0.66±0.13 | 0.606 | 0.61±0.12 | 0.68±0.20 | 0.190 |
| 176 | 0.26±0.09 | 0.27±0.08 | 0.652 | 0.26±0.08 | 0.24±0.06 | 0.441 |
| 177 | 0.46±0.10 | 0.48±0.08 | 0.360 | 0.45±0.10 | 0.40±0.08 | 0.155 |
| 178 | 0.60±0.13 | 0.60±0.13 | 0.940 | 0.56±0.11 | 0.59±0.14 | 0.517 |
| 179 | 0.44±0.08 | 0.44±0.08 | 0.976 | 0.43±0.08 | 0.47±0.08 | 0.098 |
| 180 | 0.59±0.11 | 0.60±0.11 | 0.681 | 0.54±0.09 | 0.57±0.14 | 0.481 |
| 181 | 0.28±0.11 | 0.29±0.10 | 0.517 | 0.32±0.13 | 0.27±0.06 | 0.227 |
| 182 | 0.28±0.10 | 0.26±0.08 | 0.532 | 0.27±0.10 | 0.26±0.05 | 0.675 |
| 183 | 0.28±0.06 | 0.28±0.05 | 0.988 | 0.31±0.14 | 0.28±0.05 | 0.478 |
| 184 | 0.65±0.16 | 0.62±0.13 | 0.553 | 0.60±0.15 | 0.73±0.22 | 0.058 |
| 185 | 0.57±0.14 | 0.54±0.13 | 0.362 | 0.51±0.12 | 0.51±0.09 | 0.851 |
| 186 | 0.46±0.15 | 0.43±0.08 | 0.300 | 0.54±0.15 | 0.42±0.11 | 0.041^*^ |
| 187 | 0.30±0.11 | 0.33±0.11 | 0.292 | 0.34±0.11 | 0.33±0.08 | 0.851 |
| 188 | 0.53±0.10 | 0.58±0.11 | 0.058 | 0.51±0.14 | 0.48±0.09 | 0.479 |
| 189 | 0.29±0.10 | 0.29±0.07 | 0.932 | 0.29±0.10 | 0.30±0.04 | 0.631 |
| 190 | 0.65±0.15 | 0.63±0.13 | 0.521 | 0.59±0.11 | 0.69±0.18 | 0.037^*^ |
| 191 | 0.42±0.11 | 0.47±0.13 | 0.120 | 0.44±0.14 | 0.39±0.17 | 0.346 |
| 192 | 0.48±0.13 | 0.54±0.17 | 0.087 | 0.48±0.17 | 0.43±0.09 | 0.363 |
| 193 | 0.56±0.16 | 0.53±0.13 | 0.426 | 0.52±0.16 | 0.56±0.12 | 0.455 |
| 194 | 0.23±0.04 | 0.25±0.04 | 0.298 | 0.22±0.05 | 0.23±0.06 | 0.849 |
| 195 | 0.43±0.10 | 0.42±0.08 | 0.556 | 0.47±0.10 | 0.48±0.08 | 0.761 |
| 196 | 0.40±0.09 | 0.39±0.07 | 0.798 | 0.39±0.07 | 0.39±0.08 | 0.985 |
| 197 | 0.27±0.06 | 0.26±0.05 | 0.613 | 0.28±0.09 | 0.28±0.05 | 0.918 |
| 198 | 0.57±0.09 | 0.57±0.09 | 0.717 | 0.57±0.08 | 0.58±0.08 | 0.668 |
| 199 | 0.52±0.15 | 0.52±0.15 | 0.984 | 0.54±0.19 | 0.62±0.16 | 0.222 |
| 200 | 0.45±0.08 | 0.48±0.07 | 0.090 | 0.44±0.08 | 0.44±0.06 | 0.936 |
| 201 | 0.46±0.08 | 0.47±0.09 | 0.459 | 0.46±0.07 | 0.44±0.07 | 0.567 |
| 202 | 0.45±0.09 | 0.45±0.09 | 0.911 | 0.44±0.07 | 0.43±0.08 | 0.508 |
| 203 | 0.55±0.13 | 0.52±0.12 | 0.316 | 0.50±0.12 | 0.51±0.08 | 0.885 |
| 204 | 0.33±0.12 | 0.31±0.06 | 0.538 | 0.31±0.09 | 0.30±0.07 | 0.809 |
| 205 | 0.50±0.14 | 0.50±0.17 | 0.954 | 0.53±0.17 | 0.58±0.15 | 0.403 |
| 206 | 0.20±0.04 | 0.22±0.05 | 0.086 | 0.21±0.07 | 0.23±0.09 | 0.673 |
| 207 | 0.54±0.20 | 0.53±0.15 | 0.720 | 0.61±0.15 | 0.47±0.13 | 0.014^*^ |
| 208 | 0.30±0.09 | 0.29±0.05 | 0.507 | 0.32±0.09 | 0.31±0.10 | 0.755 |
| 209 | 0.58±0.17 | 0.58±0.18 | 0.995 | 0.57±0.19 | 0.65±0.18 | 0.253 |
| 210 | 0.60±0.16 | 0.59±0.13 | 0.806 | 0.58±0.16 | 0.54±0.16 | 0.435 |
| 211 | 0.52±0.16 | 0.54±0.19 | 0.667 | 0.50±0.21 | 0.47±0.20 | 0.709 |
| 212 | 0.39±0.10 | 0.40±0.10 | 0.583 | 0.40±0.08 | 0.38±0.07 | 0.621 |
| 213 | 0.38±0.06 | 0.36±0.05 | 0.384 | 0.37±0.06 | 0.37±0.07 | 0.938 |
| 214 | 0.54±0.14 | 0.53±0.13 | 0.754 | 0.58±0.11 | 0.50±0.13 | 0.061 |
| 215 | 0.54±0.12 | 0.53±0.09 | 0.611 | 0.48±0.08 | 0.56±0.09 | 0.009^**^ |
| 216 | 0.65±0.13 | 0.66±0.14 | 0.908 | 0.61±0.14 | 0.62±0.12 | 0.874 |
| 217 | 0.29±0.09 | 0.30±0.08 | 0.678 | 0.32±0.09 | 0.32±0.06 | 0.881 |
| 218 | 0.52±0.10 | 0.53±0.10 | 0.646 | 0.51±0.11 | 0.49±0.10 | 0.589 |
| 219 | 0.75±0.17 | 0.71±0.09 | 0.243 | 0.69±0.17 | 0.71±0.13 | 0.776 |
| 220 | 0.50±0.10 | 0.51±0.09 | 0.765 | 0.49±0.11 | 0.48±0.10 | 0.806 |
| 221 | 0.36±0.11 | 0.36±0.10 | 0.827 | 0.35±0.09 | 0.40±0.16 | 0.224 |
| 222 | 0.60±0.17 | 0.56±0.14 | 0.311 | 0.55±0.14 | 0.65±0.14 | 0.047^*^ |
| 223 | 0.57±0.13 | 0.57±0.10 | 0.919 | 0.57±0.14 | 0.53±0.14 | 0.380 |
| 224 | 0.55±0.16 | 0.52±0.14 | 0.528 | 0.52±0.15 | 0.48±0.10 | 0.507 |
| 225 | 0.48±0.08 | 0.49±0.11 | 0.672 | 0.53±0.12 | 0.44±0.11 | 0.042^*^ |
| 226 | 0.52±0.12 | 0.53±0.09 | 0.850 | 0.51±0.10 | 0.54±0.12 | 0.502 |
| 227 | 0.62±0.18 | 0.62±0.17 | 0.954 | 0.55±0.17 | 0.58±0.12 | 0.637 |
| 228 | 0.54±0.14 | 0.56±0.10 | 0.526 | 0.47±0.08 | 0.54±0.13 | 0.065 |
| 229 | 0.63±0.17 | 0.58±0.14 | 0.218 | 0.57±0.16 | 0.68±0.22 | 0.106 |
| 230 | 0.76±0.14 | 0.73±0.12 | 0.307 | 0.72±0.11 | 0.73±0.14 | 0.719 |
| 231 | 0.45±0.12 | 0.44±0.08 | 0.653 | 0.46±0.11 | 0.41±0.11 | 0.213 |
| 232 | 0.49±0.09 | 0.49±0.08 | 0.870 | 0.47±0.10 | 0.51±0.09 | 0.255 |
| 233 | 0.59±0.14 | 0.57±0.15 | 0.447 | 0.55±0.14 | 0.62±0.15 | 0.157 |
| 234 | 0.49±0.13 | 0.50±0.13 | 0.673 | 0.46±0.14 | 0.42±0.15 | 0.368 |
| 235 | 0.33±0.09 | 0.32±0.07 | 0.766 | 0.35±0.09 | 0.34±0.08 | 0.741 |
| 236 | 0.61±0.11 | 0.64±0.13 | 0.300 | 0.64±0.10 | 0.62±0.15 | 0.733 |
| 237 | 0.62±0.14 | 0.61±0.13 | 0.703 | 0.58±0.13 | 0.62±0.19 | 0.405 |
| 238 | 0.30±0.07 | 0.32±0.06 | 0.273 | 0.32±0.07 | 0.38±0.13 | 0.079 |
| 239 | 0.48±0.10 | 0.52±0.11 | 0.177 | 0.45±0.09 | 0.43±0.10 | 0.598 |
| 240 | 0.44±0.09 | 0.47±0.10 | 0.179 | 0.47±0.10 | 0.43±0.12 | 0.302 |
| 241 | 0.59±0.18 | 0.55±0.15 | 0.321 | 0.54±0.16 | 0.54±0.13 | 0.938 |
| 242 | 0.69±0.12 | 0.69±0.11 | 0.802 | 0.64±0.09 | 0.65±0.12 | 0.838 |
| 243 | 0.55±0.15 | 0.52±0.25 | 0.636 | 0.50±0.17 | 0.53±0.11 | 0.616 |
| 244 | 0.57±0.11 | 0.59±0.10 | 0.628 | 0.57±0.10 | 0.54±0.10 | 0.483 |
| 245 | 0.31±0.07 | 0.34±0.09 | 0.070 | 0.34±0.08 | 0.35±0.08 | 0.743 |
| 246 | 0.56±0.16 | 0.60±0.18 | 0.377 | 0.51±0.20 | 0.48±0.16 | 0.667 |
| 247 | 0.48±0.08 | 0.51±0.10 | 0.283 | 0.49±0.07 | 0.52±0.15 | 0.453 |
| 248 | 0.71±0.17 | 0.74±0.22 | 0.578 | 0.68±0.12 | 0.65±0.15 | 0.537 |
| 249 | 0.61±0.09 | 0.62±0.10 | 0.613 | 0.61±0.13 | 0.56±0.07 | 0.222 |
| 250 | 0.33±0.07 | 0.35±0.07 | 0.240 | 0.35±0.08 | 0.36±0.12 | 0.762 |
| 251 | 0.54±0.16 | 0.51±0.08 | 0.452 | 0.49±0.10 | 0.52±0.11 | 0.334 |
| 252 | 0.49±0.10 | 0.53±0.08 | 0.056 | 0.52±0.10 | 0.45±0.09 | 0.039^*^ |
| 253 | 0.38±0.14 | 0.39±0.13 | 0.851 | 0.36±0.11 | 0.40±0.14 | 0.338 |
| 254 | 0.62±0.12 | 0.60±0.13 | 0.499 | 0.61±0.12 | 0.62±0.12 | 0.728 |
| 255 | 0.53±0.12 | 0.54±0.08 | 0.656 | 0.54±0.10 | 0.53±0.10 | 0.806 |
| 256 | 0.50±0.12 | 0.47±0.08 | 0.181 | 0.49±0.10 | 0.47±0.10 | 0.609 |
| 257 | 0.36±0.08 | 0.37±0.11 | 0.501 | 0.35±0.09 | 0.40±0.04 | 0.082 |
| 258 | 0.72±0.14 | 0.72±0.13 | 0.986 | 0.70±0.18 | 0.71±0.09 | 0.774 |
| 259 | 0.56±0.10 | 0.58±0.10 | 0.427 | 0.57±0.10 | 0.51±0.09 | 0.064 |
| 260 | 0.59±0.14 | 0.56±0.11 | 0.383 | 0.55±0.13 | 0.64±0.15 | 0.062 |
| 261 | 0.47±0.08 | 0.49±0.08 | 0.545 | 0.49±0.11 | 0.42±0.09 | 0.102 |
| 262 | 0.55±0.12 | 0.56±0.11 | 0.819 | 0.54±0.14 | 0.57±0.13 | 0.554 |
| 263 | 0.51±0.10 | 0.50±0.11 | 0.654 | 0.50±0.09 | 0.53±0.12 | 0.314 |
| 264 | 0.62±0.09 | 0.61±0.07 | 0.709 | 0.62±0.10 | 0.62±0.06 | 0.950 |
| 265 | 0.60±0.13 | 0.62±0.14 | 0.554 | 0.58±0.12 | 0.59±0.15 | 0.786 |
| 266 | 0.53±0.09 | 0.55±0.07 | 0.343 | 0.53±0.09 | 0.56±0.11 | 0.408 |
| 267 | 0.45±0.13 | 0.45±0.13 | 0.897 | 0.42±0.11 | 0.45±0.15 | 0.544 |
| 268 | 0.55±0.15 | 0.59±0.12 | 0.240 | 0.53±0.17 | 0.57±0.15 | 0.410 |
| 269 | 0.36±0.06 | 0.36±0.06 | 0.626 | 0.38±0.07 | 0.40±0.08 | 0.412 |
| 270 | 0.64±0.20 | 0.62±0.21 | 0.669 | 0.63±0.22 | 0.68±0.21 | 0.566 |
| 271 | 0.48±0.06 | 0.49±0.07 | 0.440 | 0.47±0.07 | 0.47±0.09 | 0.839 |
| 272 | 0.63±0.11 | 0.61±0.10 | 0.578 | 0.62±0.10 | 0.59±0.08 | 0.371 |
| 273 | 0.47±0.08 | 0.47±0.09 | 0.952 | 0.46±0.07 | 0.49±0.09 | 0.393 |
| 274 | 0.50±0.10 | 0.50±0.08 | 0.921 | 0.48±0.07 | 0.46±0.10 | 0.338 |
| 275 | 0.47±0.09 | 0.43±0.07 | 0.054 | 0.45±0.09 | 0.42±0.06 | 0.276 |
| 276 | 0.68±0.19 | 0.62±0.14 | 0.161 | 0.59±0.11 | 0.66±0.15 | 0.111 |
| 277 | 0.58±0.13 | 0.56±0.09 | 0.523 | 0.58±0.13 | 0.57±0.13 | 0.727 |
| 278 | 0.51±0.11 | 0.47±0.08 | 0.133 | 0.48±0.08 | 0.43±0.09 | 0.116 |
| 279 | 0.40±0.07 | 0.41±0.07 | 0.404 | 0.41±0.09 | 0.42±0.08 | 0.837 |
| 280 | 0.76±0.18 | 0.73±0.14 | 0.619 | 0.70±0.14 | 0.71±0.17 | 0.960 |
| 281 | 0.66±0.11 | 0.69±0.15 | 0.350 | 0.68±0.11 | 0.65±0.08 | 0.346 |
| 282 | 0.70±0.15 | 0.66±0.16 | 0.258 | 0.62±0.12 | 0.69±0.17 | 0.142 |
| 283 | 0.52±0.10 | 0.48±0.09 | 0.086 | 0.49±0.07 | 0.45±0.09 | 0.147 |
| 284 | 0.66±0.13 | 0.67±0.12 | 0.760 | 0.65±0.14 | 0.60±0.16 | 0.336 |
| 285 | 0.70±0.20 | 0.67±0.20 | 0.620 | 0.77±0.21 | 0.76±0.21 | 0.841 |
| 286 | 0.60±0.11 | 0.64±0.10 | 0.099 | 0.58±0.15 | 0.58±0.11 | 0.984 |
| 287 | 0.51±0.12 | 0.57±0.10 | 0.026^*^ | 0.50±0.09 | 0.48±0.14 | 0.768 |
| 288 | 0.57±0.14 | 0.62±0.13 | 0.151 | 0.55±0.14 | 0.56±0.13 | 0.923 |
| 289 | 0.56±0.10 | 0.56±0.08 | 0.849 | 0.51±0.10 | 0.63±0.08 | 0.001^**^ |
| 290 | 0.58±0.10 | 0.58±0.07 | 0.773 | 0.59±0.07 | 0.61±0.12 | 0.496 |
| 291 | 0.47±0.08 | 0.47±0.07 | 0.979 | 0.46±0.09 | 0.46±0.06 | 0.938 |
| 292 | 0.59±0.11 | 0.61±0.12 | 0.561 | 0.58±0.10 | 0.57±0.12 | 0.838 |
| 293 | 0.65±0.16 | 0.66±0.16 | 0.646 | 0.63±0.13 | 0.65±0.07 | 0.663 |
| 294 | 0.60±0.09 | 0.58±0.10 | 0.480 | 0.59±0.11 | 0.59±0.08 | 0.910 |
| 295 | 0.62±0.11 | 0.63±0.13 | 0.882 | 0.61±0.11 | 0.62±0.10 | 0.780 |
| 296 | 0.57±0.12 | 0.61±0.11 | 0.116 | 0.59±0.11 | 0.55±0.15 | 0.365 |
| 297 | 0.52±0.10 | 0.54±0.10 | 0.397 | 0.52±0.08 | 0.51±0.10 | 0.780 |
| 298 | 0.64±0.12 | 0.65±0.09 | 0.613 | 0.62±0.11 | 0.60±0.09 | 0.592 |
| 299 | 0.48±0.10 | 0.48±0.09 | 0.973 | 0.48±0.07 | 0.46±0.11 | 0.563 |
| 300 | 0.57±0.11 | 0.63±0.09 | 0.014^*^ | 0.58±0.10 | 0.59±0.04 | 0.685 |
| 301 | 0.39±0.07 | 0.39±0.05 | 0.854 | 0.42±0.07 | 0.41±0.06 | 0.809 |
| 302 | 0.51±0.11 | 0.55±0.10 | 0.118 | 0.53±0.14 | 0.49±0.11 | 0.402 |
| 303 | 0.52±0.10 | 0.51±0.09 | 0.638 | 0.53±0.08 | 0.53±0.10 | 0.791 |
| 304 | 0.59±0.14 | 0.64±0.15 | 0.133 | 0.63±0.11 | 0.53±0.14 | 0.035^*^ |
| 305 | 0.58±0.14 | 0.54±0.11 | 0.257 | 0.54±0.12 | 0.52±0.10 | 0.516 |
| 306 | 0.59±0.14 | 0.61±0.13 | 0.659 | 0.59±0.09 | 0.58±0.10 | 0.667 |
| 307 | 0.55±0.12 | 0.50±0.10 | 0.101 | 0.53±0.11 | 0.49±0.12 | 0.292 |
| 308 | 0.70±0.12 | 0.71±0.12 | 0.882 | 0.73±0.12 | 0.74±0.10 | 0.741 |
| 309 | 0.64±0.19 | 0.68±0.19 | 0.483 | 0.69±0.18 | 0.69±0.18 | 0.970 |
| 310 | 0.64±0.23 | 0.65±0.15 | 0.880 | 0.64±0.15 | 0.52±0.13 | 0.025^*^ |
| 311 | 0.55±0.10 | 0.53±0.10 | 0.362 | 0.55±0.09 | 0.54±0.08 | 0.553 |
| 312 | 0.59±0.14 | 0.55±0.12 | 0.218 | 0.55±0.11 | 0.53±0.10 | 0.683 |
| 313 | 0.61±0.15 | 0.60±0.13 | 0.715 | 0.60±0.13 | 0.57±0.18 | 0.566 |
| 314 | 0.54±0.11 | 0.57±0.15 | 0.325 | 0.56±0.11 | 0.56±0.09 | 0.991 |
| 315 | 0.58±0.10 | 0.55±0.10 | 0.285 | 0.57±0.08 | 0.54±0.08 | 0.349 |
| 316 | 0.60±0.16 | 0.64±0.16 | 0.276 | 0.66±0.18 | 0.50±0.11 | 0.015^*^ |
| 317 | 0.64±0.12 | 0.65±0.14 | 0.707 | 0.66±0.14 | 0.71±0.10 | 0.308 |
| 318 | 0.55±0.16 | 0.57±0.15 | 0.470 | 0.57±0.13 | 0.46±0.08 | 0.019^*^ |
| 319 | 0.62±0.16 | 0.63±0.15 | 0.705 | 0.62±0.15 | 0.52±0.09 | 0.036^*^ |
| 320 | 0.71±0.19 | 0.71±0.16 | 0.890 | 0.70±0.15 | 0.71±0.24 | 0.894 |
| 321 | 0.55±0.15 | 0.56±0.14 | 0.777 | 0.57±0.13 | 0.59±0.10 | 0.758 |
| 322 | 0.55±0.17 | 0.56±0.20 | 0.753 | 0.52±0.15 | 0.49±0.16 | 0.568 |
| 323 | 0.58±0.14 | 0.56±0.12 | 0.511 | 0.57±0.11 | 0.56±0.11 | 0.939 |
| 324 | 0.46±0.07 | 0.45±0.06 | 0.627 | 0.46±0.08 | 0.46±0.05 | 0.945 |
| 325 | 0.56±0.15 | 0.57±0.15 | 0.819 | 0.56±0.15 | 0.52±0.12 | 0.551 |
| 326 | 0.63±0.14 | 0.64±0.13 | 0.723 | 0.64±0.13 | 0.68±0.09 | 0.398 |
| 327 | 0.61±0.11 | 0.59±0.10 | 0.623 | 0.60±0.12 | 0.58±0.09 | 0.688 |
| 328 | 0.56±0.17 | 0.59±0.17 | 0.604 | 0.53±0.13 | 0.50±0.14 | 0.536 |
| 329 | 0.57±0.12 | 0.60±0.14 | 0.373 | 0.57±0.12 | 0.51±0.10 | 0.222 |
| 330 | 0.56±0.12 | 0.57±0.15 | 0.583 | 0.56±0.12 | 0.54±0.12 | 0.655 |
| 331 | 0.57±0.13 | 0.51±0.12 | 0.074 | 0.53±0.14 | 0.47±0.10 | 0.174 |
| 332 | 0.52±0.15 | 0.52±0.13 | 0.988 | 0.52±0.13 | 0.52±0.08 | 0.971 |
| 333 | 0.59±0.16 | 0.61±0.19 | 0.692 | 0.58±0.10 | 0.57±0.12 | 0.642 |
| 334 | 0.48±0.10 | 0.51±0.10 | 0.321 | 0.50±0.10 | 0.51±0.07 | 0.685 |
| 335 | 0.54±0.13 | 0.56±0.13 | 0.554 | 0.55±0.10 | 0.56±0.08 | 0.821 |
| 336 | 0.70±0.20 | 0.73±0.19 | 0.486 | 0.71±0.18 | 0.71±0.26 | 0.988 |
| 337 | 0.60±0.12 | 0.58±0.11 | 0.552 | 0.59±0.10 | 0.60±0.09 | 0.722 |
| 338 | 0.51±0.16 | 0.49±0.13 | 0.586 | 0.49±0.16 | 0.46±0.10 | 0.510 |
| 339 | 0.62±0.15 | 0.62±0.17 | 0.975 | 0.64±0.12 | 0.62±0.20 | 0.721 |
| 340 | 0.64±0.18 | 0.64±0.16 | 0.872 | 0.64±0.18 | 0.62±0.22 | 0.838 |
| 341 | 0.55±0.15 | 0.55±0.12 | 0.957 | 0.57±0.15 | 0.53±0.10 | 0.420 |
| 342 | 0.39±0.09 | 0.38±0.08 | 0.818 | 0.39±0.08 | 0.38±0.07 | 0.664 |
| 343 | 0.41±0.09 | 0.38±0.07 | 0.208 | 0.41±0.09 | 0.40±0.09 | 0.838 |
| 344 | 0.50±0.14 | 0.50±0.15 | 0.987 | 0.49±0.14 | 0.41±0.08 | 0.109 |
| 345 | 0.57±0.15 | 0.56±0.12 | 0.760 | 0.57±0.18 | 0.54±0.12 | 0.650 |
| 346 | 0.45±0.08 | 0.45±0.07 | 0.858 | 0.44±0.10 | 0.44±0.06 | 0.991 |
| 347 | 0.53±0.18 | 0.51±0.15 | 0.696 | 0.53±0.17 | 0.49±0.09 | 0.507 |
| 348 | 0.46±0.08 | 0.46±0.06 | 0.864 | 0.45±0.10 | 0.43±0.07 | 0.503 |
| 349 | 0.47±0.10 | 0.48±0.07 | 0.691 | 0.46±0.10 | 0.41±0.08 | 0.191 |
| 350 | 0.43±0.07 | 0.43±0.05 | 0.992 | 0.45±0.12 | 0.42±0.05 | 0.424 |
| 351 | 0.47±0.09 | 0.48±0.06 | 0.537 | 0.47±0.10 | 0.43±0.06 | 0.226 |
| 352 | 0.56±0.14 | 0.58±0.15 | 0.710 | 0.57±0.15 | 0.48±0.06 | 0.062 |
| 353 | 0.50±0.12 | 0.52±0.17 | 0.659 | 0.50±0.14 | 0.43±0.08 | 0.114 |
| 354 | 0.43±0.07 | 0.41±0.06 | 0.235 | 0.41±0.07 | 0.42±0.04 | 0.471 |
| 355 | 0.51±0.15 | 0.52±0.15 | 0.720 | 0.50±0.11 | 0.50±0.08 | 0.881 |
| 356 | 0.66±0.16 | 0.65±0.18 | 0.915 | 0.67±0.17 | 0.61±0.12 | 0.331 |
| 357 | 0.50±0.12 | 0.52±0.14 | 0.604 | 0.52±0.12 | 0.41±0.07 | 0.008^**^ |
| 358 | 0.56±0.12 | 0.51±0.09 | 0.044^*^ | 0.55±0.23 | 0.49±0.08 | 0.360 |

*: *p*<0.05; ***p*<0.01
